# Predictors of Gull-billed tern (*Gelochelidon nilotica*) nest survival in artificial coastal saltpans, Bohai Bay, China

**DOI:** 10.7717/peerj.10054

**Published:** 2020-10-05

**Authors:** Fuxing Wu, Weipan Lei, Huw Lloyd, Zhengwang Zhang

**Affiliations:** 1 Ministry of Education Key Laboratory for Biodiversity Sciences and Ecological Engineering, College of Life Sciences, Beijing Normal University, Beijing, China; 2 Fujian Provincial Key Laboratory of Marine Ecological Conservation and Restoration, Third Institute of Oceanography, Ministry of Natural Resources, Xiamen, Fujian, China; 3 Department of Natural Sciences, Manchester Metropolitan University, Manchester, United Kingdom

**Keywords:** Breeding ecology, Nest success, Saltpans, Waterbird conservation, Gull-billed Tern, Yellow Sea

## Abstract

**Background:**

Coastal saltpans are a common supratidal human-modified wetland habitat found within many coastal landscape mosaics. Commercial salt production and aquaculture practices often result in the creation of exposed coastal substrates that could provide suitable breeding habitat for waterbird populations; however, few studies have quantified waterbird breeding success in these artificial wetlands.

**Methods:**

Here we examine the nesting behavior of the Gull-billed tern (*Gelochelidon nilotica*) breeding in the Nanpu coastal saltpans of Bohai Bay, Yellow Sea, China over three consecutive nesting seasons (2017–2019) by using nest survival model in Program MARK.

**Results:**

The results revealed that nest survival of Gull-billed terns in coastal saltpans (0.697) was higher than previously published estimates from other regions, with an estimated daily survival rate (DSR) of 0.982 ± 0.001 (±95% CI). High nest survival was mainly attributed to low levels of human disturbances and low predation rates, while exposure to strong winds, flooding and silting were the main factors causing nest failure. Model-averaged estimates revealed that eggs laid in nests located on ‘habitat islands’ with feather or clam shell substrates were most likely to hatch. Initiation date, nest age, clutch size and quadratic effects of nearest-neighbor distance, nearest distance to road and nearest distance to water were all significant predictors of nest success, but the nest survival declined overall from 2017 to 2019 due to the degradation and loss of breeding habitat anthropogenically caused by rising water levels.

**Discussion:**

Coastal saltpans represent an alternative breeding habitat for the Gull-billed tern populations in Bohai Bay, but conservation management should prioritize flood prevention to improve the extent and quality of breeding habitat, concurrent with efforts to create further ‘habitat islands’ with suitable nesting substrate.

## Introduction

Human disturbance and land-use changes driven by tidal reclamation have resulted in the loss and degradation of coastal wetland ecosystems globally (Delany et al., 2009; Hoegh-Guldberg & Bruno, 2010). These losses pose serious threats to waterbird populations dependent on these wetlands for staging, non-breeding, or breeding habitat (Warnock et al., 2002; Kirwan & Megonigal, 2013; Ma et al., 2014). Concurrently, artificial wetlands are rapidly expanding, caused by commercial enterprises such as salt production, and are creating a combined landscape mosaic of both anthropogenic and natural origins that can provide alternative or supplemental breeding and foraging habitats for waterbird species (Houston et al., 2012; Bluso-Demers et al., 2016; Rocha et al., 2017; Lei et al., 2018; Barnagaud et al., 2019).

Coastal saltpans represent one of the most widespread supratidal human-modified wetland habitats (Sripanomyom et al., 2011) used for commercial production of sea-salt (Harvey et al., 1988), but these areas also host a number of alternative economic activities linked to extensive aquaculture, such as artisanal fisheries and commercial production of artemia shrimp (De Medeiros Rocha et al., 2012). The process of salt production involves the construction of equal sized bodies of water by artificially pumping seawater into large coastal saltpans. Studies have shown that these artificial water bodies can support proportions of migratory waterbird populations in many coastal regions due to their proximity to migratory fly-ways (e.g.,  Takekawa, Lu & Pratt, 2001; Li et al., 2013; Lei et al., 2018; Barnagaud et al., 2019) by providing important foraging areas and protected roosting sites (e.g.,  Sripanomyom et al., 2011; Rocha et al., 2017). Salt production also creates ‘habitat islands’ that can serve as breeding habitats for a small number of waterbird species (Britton & Johnson, 1987; Yasué, Patterson & Dearden, 2007; Chokri & Selmi, 2011). This has led some authors to suggest that adequate conservation management of saltpans could provide complementary habitat to help alleviate some of the impact on waterbirds caused by the loss of natural wetlands (e.g., Masero, 2003; Béchet et al., 2009; Rocha et al., 2017).

Distinct from natural wetlands, waterbirds breeding in saltpans are confronted with different challenges (i.e., instantaneous water level adjustment, disturbance due to commercial activities of salt collection, little vegetation and soil substrates easily flooded by heavy rainfall). Salt concentrations found in saltpans, often exceed 300 mg/L, and can only be tolerated by a handful of salt-adapted species, such as Pied avocet (*Recurvirostra avosetta*), Black-winged stilt (*Himantopus himantopus*), Black-tailed godwit (*Limosa limosa*) and Marsh sandpiper (*Tringa stagnatilis*) (Walmsley, 1999; Lei et al., 2018). Few studies have examined the breeding success of waterbirds in these artificial wetlands (Martin, 1987; Tavares et al., 2008; Que et al., 2015; Rocha et al., 2016).

Saltpans have become a common component of coastal wetland landscape in Bohai Bay, in the Yellow Sea of China (Wang, Xu & Zhu, 2015; Lei et al., 2018). These highly dynamic coastal wetland landscapes provide essential stop-over/staging and wintering habitats for numerous migratory bird species along the East-Asian Migratory Fly-Way (Yang et al., 2011; MacKinnon, Verkuil & Murray, 2012; Melville et al., 2016). These wetlands also play a critical role in geochemical cycles (Twilley, Chen & Hargis, 1992), regulate regional climate (Day et al., 2008), and act as a carbon sink (Smith, DeLaune & Patrick Jr, 1983a; Hopkinson, Cai & Hu, 2012). Intertidal habitats along the Yellow Sea are disappearing at an alarming rate and have decreased by almost 65% over the past 50 years due to land reclamation and development (Que et al., 2015; Ma et al., 2014; Murray et al., 2014), with extreme consequences for waterbird populations (Amano et al., 2010; MacKinnon, Verkuil & Murray, 2012). Urban areas such as the city of Tianjin have a long-established salt production industry, with the area of commercial saltpans in the region increasing from 60.0 km^2^ in 1979 to approximately 457.8 km^2^ in 1988 at the expense of natural wetland habitat (Wang, Xu & Zhu, 2015). While saltpans and other artificial wetlands are known to provide foraging habitat for a number of migratory waterbird species (e.g.,  Li et al., 2013; Lei et al., 2018), little is known about whether these artificial habitats are able to provide alternative breeding habitat for birds within this landscape mosaic (Yasué, Patterson & Dearden, 2007; Chokri & Selmi, 2011; Que et al., 2015).

The Gull-billed tern *Gelochelidon nilotica* is a middle-sized waterbird species with a widespread global distribution, with localized breeding populations found on six continents (Møller, 1982; Molina & Erwin, 2006). The species occupies a variety of habitats that include inland freshwater or saltwater lakes, rivers, and marshes (Snow & Perrins, 1998; Zhao, 2001), although it remains poorly studied across much of its range and may have experienced population declines (Erwin et al., 1999; Molina & Erwin, 2006). Consequently it is currently listed as conservation concern in Europe, Africa and North America (Kushlan et al., 2002; Sánchez et al., 2004; Molina & Erwin, 2006; Staneva & Burfield, 2017). Gull-billed tern is an opportunistic dietary generalist that uses both terrestrial and aquatic resources in wetland mosaics that mainly includes lizards, flying insects, estuarine fish, and both marine and estuarine crab species (Bogliani et al., 1990; Stienen, Brenninkmeijer & Klaassen, 2008; Albano et al., 2011; Goodenough, 2013). Typically the species breeds in loose aggregations generally near water with suitable foraging habitats that include estuaries, deltas, lagoons, inland lakes, rivers, marshes, but also artificial wetlands such as aquaculture ponds and dikes of salt production ponds (Molina & Erwin, 2006; Barati, Etezadifar & Esfandabad, 2012; Goodenough & Patton, 2020).

Two subspecies are known to breed within China—*G. n. nilotica* has been recorded breeding in Xinjiang and *G. n. addenda* has been recorded breeding south of Liaoning Province and our study area (Zhao, 2001; Zheng, 2017). In this study, we report on the nesting success of the Gull-billed tern breeding in the Nanpu coastal saltpan habitats of Bohai Bay. We identify the predictors of nest success from three consecutive breeding seasons and determine the alternative of these artificial wetlands as breeding sites for the species within the wetland mosaics of northern Bohai Bay based on previous work on the breeding success of this species (Erwin et al., 1999; Barati, Etezadifar & Esfandabad, 2012; Villegas et al., 2013; Windhoffer et al., 2017).

## Materials & Methods

### Study sites

**Figure 1:**
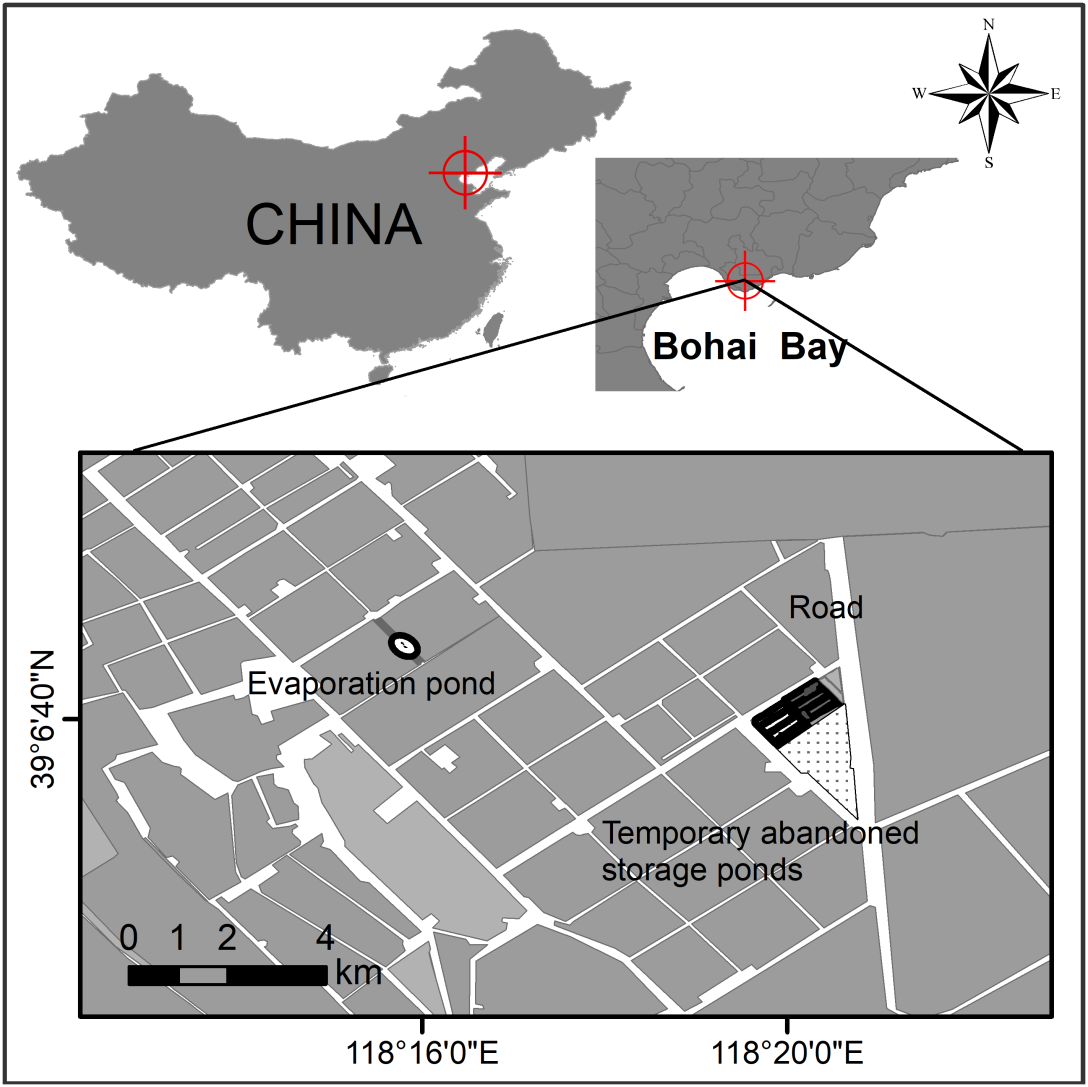
Study site at the Nanpu wetland, Bohai Bay, China. One breeding site of Gull-billed tern (*Gelochelidon nilotica*) was found at an evaporation pond and the other was located at the temporary abandoned storage pond area.

Study sites were located in northern Bohai Bay, Yellow Sea, China (Fig. 1). The Nanpu saltpans are one of the largest (290 km^2^) saltpan complexes in the world (Lei et al., 2018), consisting of storage, evaporation and crystallization ponds, which are separated by dikes. Both the dikes and some small newly created ‘habitat islands’ in the salt ponds provide breeding sites for waterbird species such as Pied avocet, Black-winged stilt, Kentish plover (*Charadrius alexandrinus*), Common tern (*Sterna hirundo*), Little tern (*Sternula albifrons*) and Gull-billed tern.

The breeding sites of Gull-billed tern are located on one sand-shell ‘island’ situated in the middle of an evaporation pond, extending on dikes and bottom of a temporary abandoned storage pond area (Fig. 1). These sites are devoid of vegetation cover and isolated from land by water. The sand-shell ‘island’ was used by breeding Gull-billed tern only in 2017 when the ‘island’ was approximately 0.002 km^2^. Rising water levels in 2018 (by 20 cm) and 2019 (by 35 cm) greatly reduced areas available within the location for breeding to the extent that no pairs bred on the ‘island’ during those years. The temporary abandoned storage pond area consisted of muddy ground, with some clam shells and gravel evident in parts of the pond dikes, and included three dried storage ponds at approximately 0.1 km^2^ each with several 10-35 m wide pond dikes that connected several salt ponds (Fig. 1). The three dried storage ponds were re-used as storage ponds in 2019 and flooded due to the water level rising by 50 cm compared with that in 2018. Consequently, all the Gull-billed terns bred on the pond dikes or ‘islands’.

### Data collection

This study was conducted between 2017 and 2019 at the Nanpu saltpans from May to July. Gull-billed tern nests were systematically searched by experienced researchers in the salt ponds and pond dikes from early May through early July during each breeding season. For some of the bigger salt ponds, we used an unmanned aerial vehicle (UAV) to locate and record the tern breeding sites, from a height of approximately 80 m.

Once the potential breeding colony was discovered (i.e., presence of adult pairs), we conducted systematic ground-based searches for nests. Once a nest was found, its location was recorded using a handheld GPS (Garmin, MAP63csx) and photographed after it was individually marked with a small wooden peg positioned at a distance of approximately 50 cm from the nest. Each egg was marked with a nontoxic marker to indicate egg and nest number. We measured the width and length of the eggs with a Vernier caliper and eggs were weighed by using an electronic balance only when they were confirmed to have been laid less than three days prior. Clutch initiation date was extrapolated either by the egg flotation method (Mayfield, 1961; Hensler & Nichols, 1981; Que et al., 2015) or backdating from estimated egg-laying or hatching dates assuming a two-day egg laying interval for each egg (Sears, 1978). We classified the microhabitat of nest site as mud, clamshell, feather or gravel.

Nest fate was monitored at 3–4 day intervals (Lombard, Collazo & McNair, 2010; Nefas et al., 2018) until it was recorded as failed or 2–3 days before the hatching date, after which we visited the nests at 2-day intervals to check for signs of hatching until the end of the nesting attempt (i.e., hatching or failure). Gull-billed tern chicks leave the nest within the first few days following hatching and the eggshells are removed after hatching by the parents or wind (Sears, 1978). Consequently, we considered nest fate as ‘successful’ if ≥1 egg showed evidence of hatching (one recently hatched chick in the nest or nearby) or evidence of imminent hatching (cracked or drilled eggshell). We considered a nest to have ‘failed’ when; (1) eggs disappeared before the expected hatching date; (2) the egg was damaged, blown/removed of the nest scrape, adhered to the mud or remained unhatched longer than one week of the presumed date of hatching, with no evidence of predation or flooding; (3) the nest was flooded, i.e., the bottom of the nest was wet, or the eggs were totally or partially submerged in water; or (4) eggs were found collected by local people by analyzing the photos and videos from the infrared camera (Loreda, L710); (5) the clutch was intact, but the eggs were cold unattended for ≥2 visits. These nests were classified nest failure reason as unknown. We dated nest failure to the first date we found the eggs cold and unattended. When the nest did not meet the above criteria for success or failure, we classified fate as ‘unknown’ and did not include these nests in the subsequent nest survival analyses.

### Data analyses

We used the nest survival model in Program MARK 9.0 (Dinsmore, White & Knopf, 2002; Cooch & White, 2014) to estimate daily nest survival (DSR) and to evaluate factors that influenced DSR (Que et al., 2015). Program MARK ranks candidate models based on Akaike’s information criterion corrected for small sample sizes (AIC_c_; Burnham & Anderson, 2002). Nesting season dates were standardized among years by using the date that the first nest was found in any year as the first day of the nesting season and, similarly, the latest hatching or failure date in any year as the last day of the nesting season (Moynahan et al., 2007). We included the following variables in the nest survival models: (1) the day a nest was found, (2) the last day the nest was checked when alive, (3) the last day the nest was checked, and (4) nest fate (i.e., survived or failed). We examined the influence of ten environmental and temporal covariates on DSR and their interactions: (1) nest age since estimated initiation; (2) nest habitat type, i.e., abandoned salt ponds, salt pond dikes, ‘island’ in the evaporation pond or ‘islands’ in abandoned salt ponds; (3) substrate of the nest, i.e., mud, clamshell, feather or gravel; (4) clutch size; (5) nearest-neighbor distance to a conspecific nest; (6) the nearest distance between the nest and water edge; (7) the relative height (elevation) of the nest site above the water, i.e., low, middle or high; (8) density of Gull-billed Tern nests within a 100 m radius; (9) the nearest distance to the road, and (10) the year of breeding. We ranked all the competing models and used all the selected candidate models (ΔAICc < 2) to computed average weights (*wi*) for each covariate (Johnson & Omland, 2004).

The apparent nest survival and average nest survival were calculated as follows: apparent nest survival = numbers of nests hatching success/ (numbers of nests hatching success + numbers of nests hatching failure); average nest survival = (daily nest survival)^incubation period^ (Que et al., 2015). Spatial covariates (i.e., nearest-neighbor distance to a conspecific nest, the nearest distance between the nest to the road, the nearest distance between the nest and water edge, the density of nests within a 100 m radius) were estimated by using ArcGIS 10.3 (ESRI, Redlands, CA) and the quadratic effects of the continuous covariates were also tested.

### Field permit statement

Permission to access field sites were provided by Forestry Department of Hebei Province in 2017 and Luannan County Natural Resources and Planning Bureau for work conducted in 2018 and 2019.

## Results

We identified a 60-day nesting season for Gull-billed tern at our Bohai Bay study site, which began on 9 May and ended on 7 July, resulting in 59 daily intervals for estimating the daily nest survival. More than 436 nests were found at a temporary abandoned storage pond area in 2017, which were not monitored regularly and were not then used in the subsequent analyses. In total, we monitored 1,234 nests and determined the fate of 1,143 nests. Of these, 797 nests were successful, and 346 failed, resulting in an apparent nest survival of 69.7%.

In our constant model, we estimated daily nest survival as 0.982 ± 0.001 [±95% confidence interval (CI)]. Using the data of 107 monitored nests whose laying and hatching dates were precisely known, we found that the mean (± SE) incubation period lasted 23.9 ± 0.1 days. Thus, we assumed that the average incubation period of Gull-billed tern at Bohai Bay was 24 days, which resulted in average nest survival of 64.7%.

The main causes of nest failure were related to weather conditions and anthropogenic disturbances, i.e., nests destroyed by strong wind (33%, *n* = 114), or anthropogenic flooding due to water pumping activity for salt production (30.9%, *n* = 107). Other causes of nest failure were eggs disappeared before the expected incubation period (5.5%, *n* = 19), eggs collection by local people (3.5%, *n* = 12), eggs still being incubated by parent birds after more than one week of the presumed date of hatching (2.9%, *n* = 10), predation (1.2%, *n* = 4) and unknown for 76 (23.1%) of the observed nests.

Nine candidate models of environmental and temporal covariates describing variation in DSR had ΔAIC_c_ <2 (Table 1). Consequently, averaged covariates were modeled according to their respective AIC_c_ weight (*w*_i_) to obtain robust estimates of the model parameters. After model averaging, the most important variables explaining the variation in DSR (i.e., *w*_i_ = 1) were nest initiation date, nest age, clutch size, habitat type, nest substrate type, breeding year, and the quadratic effects of nest initiation date, clutch size, nearest-neighbor distance, and nearest distance to road (Table 2). The quadratic effects of the nearest distance to water (*w*_i_ = 0.899) and the linear effects of nearest distance to road (*w*_i_ = 0.767) were also significant predictors but had lower AIC_c_ weights than the aforementioned variables (Table 2).

**Table 1:** Top nine models (ΔAICc < 2) showing the different environmental and habitat predictors of daily nest survival of the Gull-billed tern (*Gelochelidon nilotica*) breeding in coastal saltpans, Nanpu, Bohai Bay, China.

Model^a^	ΔAICc^b^	*w*_i_	K
T+T^2^+NAge+Eggs+Eggs^2^+Substrate+Habitat+ Disnest^2^+Disroad+Disroad^2^+Diswater^2^+Year	0.000	0.057	18
T+T^2^+NAge+Eggs+Eggs^2^+Substrate+Habitat+ Disnest^2^+Disroad^2^+Diswater^2^+Year	0.733	0.039	17
T+T^2^+NAge+Eggs+Eggs^2^+Substrate+Habitat+ Disnest^2^+Disroad+Disroad^2^+Diswater+Diswater^2^+Year	1.323	0.029	19
T+T^2^+NAge+Eggs+Eggs^2^+Substrate+Habitat+ Disnest^2^+Disroad+Disroad^2^+Diswater+Year	1.492	0.027	18
T+T^2^+NAge+Eggs+Eggs^2^+Substrate+Habitat+Nest100+ Disnest^2^+Disroad+Disroad^2^+Diswater^2^+Year	1.658	0.025	19
T+T^2^+NAge+Nage^2^+Eggs+Eggs^2^+Substrate+Habitat+ Disnest^2^+Disroad+Disroad^2^+Diswater^2^+Year	1.753	0.024	19
T+T^2^+NAge+Eggs+Eggs^2^+Substrate+Habitat+Disnest^2^+ Disroad^2^+Diswater+Diswater^2^+Year	1.835	0.023	18
T+T^2^+NAge+Eggs+Eggs^2^+Substrate+Habitat+Nest100^2^+ Disnest^2^+Disroad+Disroad^2^+Diswater^2^+Year	1.910	0.022	19
T+T^2^+NAge+Eggs+Eggs^2^+Substrate+Habitat+Disnest+ Disnest^2^+Disroad+Disroad^2^+Diswater^2^+Year	1.998	0.021	19

**Notes.**

AICc Akaike’s information criterion for small sample sizes *w*_*i*_ AICc weight

a T, nest initiation date; NAge, nest age; Eggs, clutch size; Disnest, nearest-neighbor distance to conspecific nest; Disroad, nearest distance to the road; Diswater, nearest distance to water; Nest100, density nests within a 100 m radius

b AICc of the top model was 2367.3

**Table 2:** Model-averaged parameter estimates and descriptive statistics for predicting the influence of different environmental and habitat characteristics on the daily nest survival of the Gull-billed tern (*Gelochelidon nilotica*) breeding in coastal saltpans.

Parameter	*β*	SE	Lower CI 95%	Upper CI 95%	*w*_i_
Intercept	−0.594	0.969	−2.494	1.305	1.000
T	0.148	0.024	0.101	0.195	1.000
T^2^	−0.003	0.000	−0.004	−0.002	1.000
Nage	−0.040	0.014	−0.068	−0.012	1.000
Eggs	3.228	0.414	2.417	4.039	1.000
Eggs^2^	−0.608	0.100	−0.805	−0.412	1.000
Mud	−0.880	0.255	−1.379	−0.380	1.000
Shell	0.733	0.344	0.059	1.407	1.000
Feather	0.768	0.286	0.208	1.328	1.000
Pond	−1.672	0.441	−2.536	−0.808	1.000
Way	−0.956	0.432	−1.803	−0.109	1.000
Island	−1.071	0.782	−2.604	0.462	1.000
Disnest^2^	0.000	0.000	0.000	0.000	1.000
Disroad^2^	0.000	0.000	0.000	0.000	1.000
2017	2.602	0.774	1.085	4.119	1.000
2018	1.192	0.158	0.881	1.502	1.000
Diswater^2^	0.000	0.000	0.000	0.000	0.899
Disroad	0.002	0.001	−0.001	0.005	0.767
Diswater	0.002	0.009	−0.016	0.020	0.296
Nest100	0.000	0.002	−0.004	0.003	0.093
Nage^2^	0.000	0.000	−0.001	0.000	0.089
Nest100^2^	0.000	0.000	0.000	0.000	0.082
Disnest	0.000	0.003	−0.006	0.006	0.079

**Notes.**

T, nest initiation date; Nage, nest age; Eggs, clutch size; Disnest, nearest-neighbor distance to conspecific nest; Disroad, the nearest distance to the road; Diswater, nearest distance to water; Nest100, density nests within a 100 m radius and CI, Confidence interval

**Figure 2:**
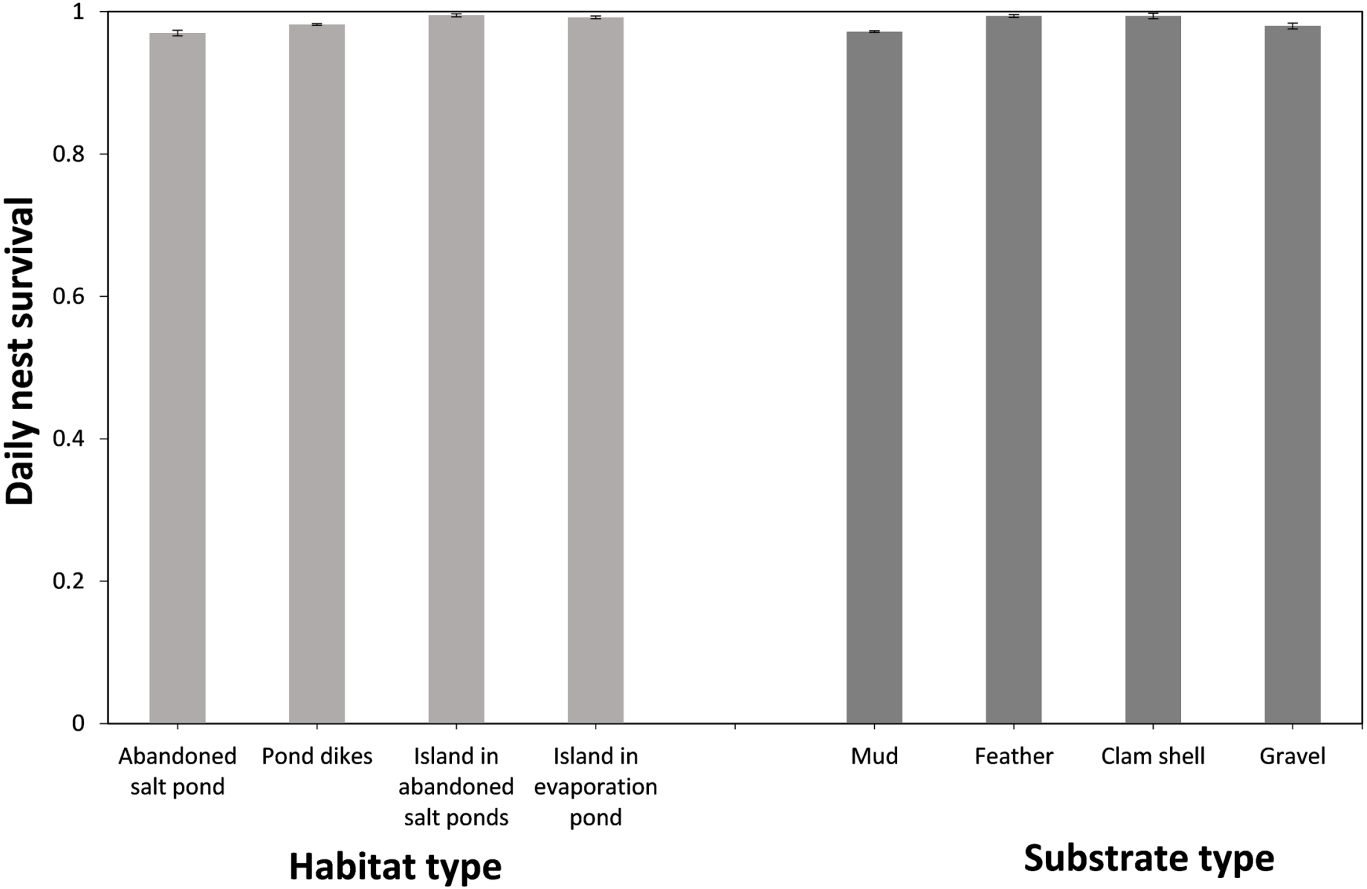
Daily nest survival [±95% confidence interval (CI)] of the Gull-billed tern (*Gelochelidon nilotica*) among the different nesting habitats and nest substrates in the Nanpu wetland, Bohai Bay, China.

Based on model-averaged estimates, the selection of appropriate nesting substrate and position was the main factor influencing DSR, with eggs laid in nests on ‘islands’ with feather or clamshell substrate most likely to hatch (Table 2, Fig. 2). More than half of the nests with known fates identified at our study site were located at pond dikes in the abandoned storage pond area (73.8%, *n* = 844), while the remaining nests were located in bottom of abandoned salt ponds (12.5%, *n* = 143), ‘islands’ in abandoned storage ponds (7.3%, *n* = 83), and ‘island’ in the evaporation pond (6.4%, *n* = 73). Nests built on the abandoned salt ponds had the lowest success among the four habitats (apparent 0.559, DSR 0.970 ± 0.004; Fig. 2), and within nest survival was highest on ‘islands’ in abandoned ponds (apparent 0.918, DSR 0.995 ± 0.002; Fig. 2), followed by the ‘island’ in the evaporation pond (apparent 0.886, DSR 0.992 ± 0.002; Fig. 2) and pond dikes (apparent 0.687, DSR 0.982 ± 0.001; Fig. 2). We found 641 nests built on a mud substrate, 323 nests on a feather substrate, 129 nests on a clamshell substrate and 50 nests on a small gravel substrate. The nests on the mud substrate had the lowest survival (apparent 0.575, DSR 0.972 ± 0.002; Fig. 2) compared to those on gravel (apparent 0.608, DSR 0.980 ± 0.004; Fig. 2), clamshell (apparent 0.878, DSR 0.994 ± 0.002; Fig. 2) or feather substrates (apparent 0.879, DSR 0.994 ± 0.001; Fig. 2).

**Figure 3:**
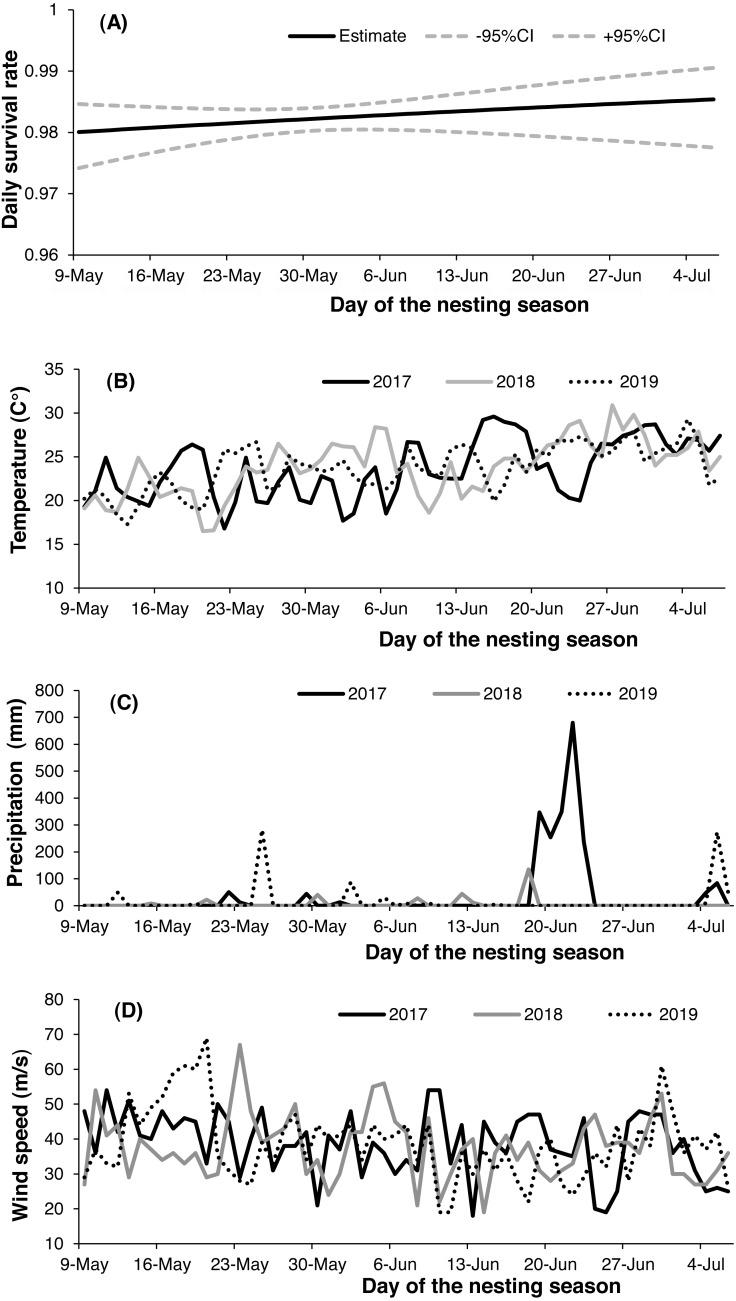
Variation in daily nest survival [±95% confidence interval (CI)] of the Gull-billed Tern (*Gelochelidon nilotica*) nests in the Nanpu wetland, Bohai Bay (A), with varying daily temperatures (B), wind speeds (C) and precipitation (D) throughout the three consecutive nesting seasons (2017–2019).

**Figure 4:**
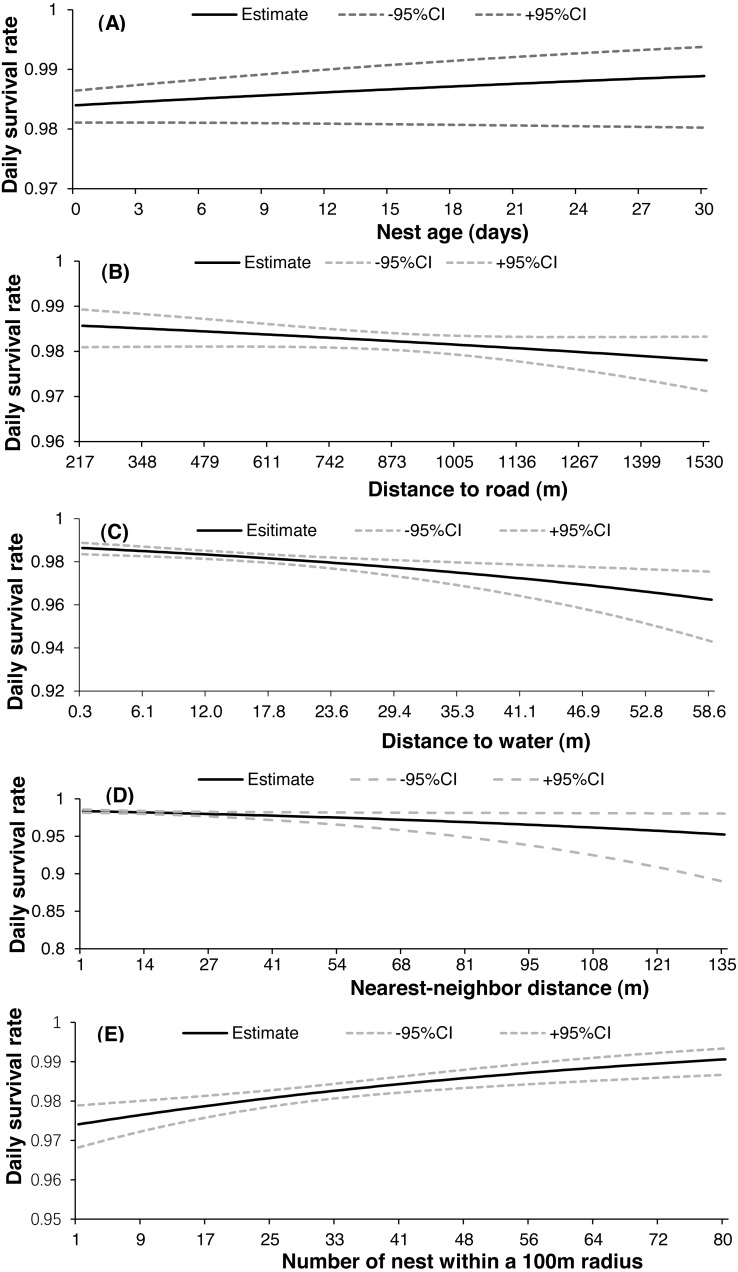
Daily nest survival [±95% confidence interval (CI)] of the Gull-billed tern (*Gelochelidon nilotica*) in the Nanpu wetland, Bohai Bay, in relation to the age of nest (A), distance to the nearest road (B), distance to nearest water (C), distance to nearest-neighbor (D), and the number of nests within a 100 m radius (E).

Both 2-egg and 3-egg clutches showed a similar relationship with DSR, and both were higher than single-egg clutches. There were only two nests with 4-egg clutches in our study, and both failed (eggs were still being incubated by parent birds after more than one week of the presumed date of hatching). Nest survival increased slightly throughout the nesting season (Fig. 3A) but decreased from 2017 (apparent 0.886, DSR 0.994 ± 0.002) to 2019 (apparent 0.547, DSR 0.973 ± 0.002). There was noticeable variation in daily temperature, wind speed and precipitation throughout each breeding season, with mean daily temperature generally increasing across the incubation period for all nesting seasons (Fig. 3B), but mean daily wind speed showed the opposite trend (Fig. 3C); in addition, the highest level of precipitation was recorded in 2017 (Fig. 3D). The relationship between nest age and DSR showed slightly increasing trend (Fig. 4A), and nest survival was relatively higher when the nest was located closer to the nearest road (Fig. 4B), with the same trend evident between distance to nearest water body and DSR (Fig. 4C). Nest survival was relatively higher when nearest-neighbor distance was low (Fig. 4D) and was also higher with increasing nest density (Fig. 4E).

## Discussion

Our results show that the Nanpu saltpans provide suitable nesting habitats for the population of Gull-billed tern in Bohai Bay. The number of nesting pairs we counted at the breeding sites was larger than those reported in other Gull-billed tern studies from North America and Iran (Palacios & Mellink, 2007; Erwin et al., 1999; Barati, Etezadifar & Esfandabad, 2012; Windhoffer et al., 2017). In addition, our estimates of nest survival are among the highest reported for this species worldwide, including those reported from natural wetland habitats in Iran (apparent nest survival 0.46 (*n* = 67); Barati, Etezadifar & Esfandabad, 2012), wetlands in Virginia, USA (apparent nest survival 0.501, *n* = 433; Erwin et al., 1999) and Isles Dernieres Barrier Islands Refuge, Louisiana, USA (apparent nest survival 0.56, *n* = 191; Windhoffer et al., 2017), which suggests that there is conspecific variation in nest survival and susceptibility to different forms of disturbance associated with human activities and predation (Que et al., 2015; Windhoffer et al., 2017).

Terns are considered particularly vulnerable to disturbance (Scarton, Valle & Borella, 1994; Catry et al., 2004), so the nest survival of Gull-billed tern in our study is encouraging as terns appear to select nesting locations in the center of evaporation ponds or abandoned storage ponds with minimal production/cultivation or other human activities. However, egg poaching by local people, which caused 12 nest failures of the Gull-billed tern at one dike in 2019, is a widespread and common phenomenon along the coastal regions of Bohai Bay, as reported for Kentish plover (Que et al., 2015) and Pied avocet (Lei, 2017). Increased patrolling of nesting areas by wildlife conservation staff throughout artificial and natural wetlands in the region during the breeding season should be a conservation management priority.

Nest predation was rarely recorded during our study where the breeding sites of Gull-billed tern were isolated, which may have served as protection against terrestrial mammalian predators, such as dogs, foxes, and rats, as observed in other studies (e.g., Nelson, 1979; Hamer, Schreiber & Burger, 2002). Although the eggs and chicks of Gull-billed tern have been reported to be prey for other bird species such as Great horned owl (*Bubo virginianus*), Barn owl (*Tyto alba*), Short-eared owl (*Asio flammeus*), Yellow-legged gull (*Larus cachinans*) and Black-headed gull (*L. ridibundus*) (Sánchez & Blasco, 1986; Sánchez et al., 2004), we suspect that the effect of avian predators in our study was somewhat minimal because only a Peregrine falcon (*Falco peregrinus*) was observed on a single occasion during our surveys.

Previous studies have found no relationship between nest site selection and hatching success for Gull-billed tern with elevation or habitat type (e.g., Erwin et al., 1998; Rounds, Erwin & Porter, 2004; Barati, Etezadifar & Esfandabad, 2012). However, our models revealed that the position of the nest was a significant predictor of DSR, and we found the lowest nest survival at abandoned salt ponds where the elevation of the nest site above the water was relatively low, and flooding with pronounced fluctuations in water-levels accounted for most nest losses, as reported in other study areas (e.g., Biber, 1993; Windhoffer et al., 2017). Pond banks and ‘islands’ in the ponds were at a relatively high level above the water, which prevented immersion even in stormy weather or during the water pumping period for salt production.

Micro-habitat features could also play an important role in nest survival (Colwell et al., 2011; Hardy & Colwell, 2012; Que et al., 2015). All nests of the Gull-billed tern at Nanpu were constructed in areas that were devoid of vegetation which we found to be a significant predictor of nesting success. The survival of nests constructed on shell, rock or feather materials was higher than that of nests built on other substrates. At Nanpu, micro-habitat features may minimize reproductive failure caused by flooding and silting or by damaged from strong winds, but further evidence is required. Daily precipitation is known to be one of the most important factors influencing nest performance (Halse & Jaensch, 1989; Que et al., 2015). In our study area, precipitation had minimal influence on tern nest survival because the noticeable peak in daily precipitation occurred toward the latter half of the breeding season when there were only a few active tern nests.

Temporal variation in nest survival across the incubation period and among breeding seasons is a common phenomenon in many avian populations (Wilson, Martin & Hannon, 2007; Kolada, Casazza & Sedinger, 2009; Smith & Wilson, 2010). Gull-billed tern nest survival at our study site also varied intra- and inter-annually, showing a slight increase in survival throughout the nesting season but inter-annually nest survival decreased from 2017 to 2019. We suspect that this may have been influenced in part by variation in local climate conditions at the study site and perhaps differences in the level of conservation management across the different years.

In 2018, the abandoned salt ponds were used as storage ponds and nearly half of all nests located within the abandoned salt ponds were flooded when water was pumped into the ponds. Water levels within the ponds rose by 50 cm in 2019 such that all the salt ponds and a portion of the pond dikes were completely submerged. Additionally, three days of high winds occurred during the highest egg laying period in 2019. Combined, the flooding and winds resulted in almost a quarter of all tern nests being destroyed.

## Conclusions

Artificial wetland habitats such as saltpans may become increasingly important as an alternative nesting habitat for populations of coastal waterbird species, with the continuing loss and degradation of natural coastal wetlands caused by increasing human disturbance (Rocha et al., 2016). Our work suggests that coastal saltpans could constitute an attractive alternative nesting habitat for the Gull-billed tern and highlights their poorly recognized conservation value in the Bohai Bay region of coastal China. Our results have implications for the conservation management of waterbirds primarily because conservation management should focus on improving and restoring nesting habitat quality, and regular (annual) surveys of nesting bird abundance. Future conservation plans need to prioritize measures that reduce efforts to manipulate saltpan habitat to prevent flooding and nest abandonment. Since saltpans are not usually influenced by tidal regimes and water levels are influenced either by anthropogenic means (pumping for salt production) or by a direct result of weather events, further efforts to control water-level fluctuations during the breeding season will be essential to preventing nesting habitat loss and displacement of nesting pairs. Draining abandoned evaporation ponds before the onset of the nesting season, complemented by efforts to create further artificial ‘islands’, in addition to the provision of suitable nesting substrates (e.g., shell/gravel mixtures), and increasing the width of dikes where minimal human disturbance occurs would represent a package of achievable conservation measures to enhance the reproductive success of waterbirds in the Nanpu wetlands. Meanwhile, future surveys are needed to determine what food resources may be located near the alternative nesting locations.

##  Supplemental Information

10.7717/peerj.10054/supp-1 Data S1 Raw data for analysing the nest survival of Gull-billed tern

## Additional Information and Declarations

### Field Study Permissions

The following information was supplied relating to field study approvals (i.e., approving body and any reference numbers): Permission to access field sites were provided by Forestry Department of Hebei Province in 2017 and Luannan County Natural Resources and Planning Bureau for work conducted in 2018 and 2019.

### Data Availability

The following information was supplied regarding data availability: The raw measurements are available as a Supplemental File.
